# The Relationship between Attitudes toward Death and Emotional Intelligence, Personality, Resilience, and Justice Beliefs: A Cross-Sectional Study of Midwives in Greece

**DOI:** 10.3390/ejihpe14040072

**Published:** 2024-04-21

**Authors:** Evangelos Tzamakos, Dimitra Metallinou, Antigoni Sarantaki, Maria Tigka, Aikaterini Lykeridou, Christina Nanou

**Affiliations:** 1 Department of Midwifery, Faculty of Health and Care Sciences, University of West Attica, 12243 Athens, Greece; tzamvagpal@uniwa.gr (E.T.); esarantaki@uniwa.gr (A.S.); klyker@uniwa.gr (A.L.); nanouxv@uniwa.gr (C.N.); 2 Delivery Room, General and Maternity Hospital “Helena Venizelou”, 11521 Athens, Greece; maria.tigka@gmail.com

**Keywords:** midwifery, attitudes, hospital, healthcare professionals, death

## Abstract

Midwifery practice inevitably includes miscarriages, stillbirths, and neonatal deaths. The aim of the present study was to investigate the relationship between attitudes toward death and emotional intelligence, personality, resilience, and justice beliefs among midwives in Greece. A descriptive cross-sectional study was conducted from 2020 to 2022 among 348 midwives employed in public hospitals, in regional health authorities, or as independent professionals. Research instruments included the Death Attitude Profile—Revised, the Connor–Davidson Resilience Scale, the Trait Emotional Intelligence Questionnaire—Short Form, the Eysenck Personality Questionnaire, and the Belief in a Just World scale. The results revealed that greater emotional intelligence was significantly associated with higher scores in the escape acceptance subscale. Midwives scored low on the neutral acceptance subscale (2.9 ± 0.8), with the highest score being recorded in the escape acceptance subscale (4.6 ± 1.0), which was significantly associated with greater emotional intelligence. Neuroticism was significantly associated with the death avoidance, approach acceptance, fear of death, and escape acceptance subscales. Finally, the subscale of distributive justice beliefs for self and others was significantly associated with the subscales of death avoidance and approach acceptance. These findings highlight the nuanced perspectives within the healthcare community. As we delve deeper into the complexities of end-of-life care, understanding these diverse attitudes is crucial for providing comprehensive and empathetic support to both patients and healthcare professionals.

## 1. Introduction

Midwifery is a field of healthcare focused on the well-being of the mother–child dyad during pregnancy, labor, birth, and the postpartum period. Midwives’ role is multidimensional, and their responsibilities are multifaceted, including, in brief, providing evidence-based clinical care, education, and counseling, health promotion, healthcare advocacy and policy changes, collaboration with other healthcare professionals (HPs) and referral to specialists, and emotional and psychological support [1]. Unfortunately, despite the best efforts of midwives, death remains an integral aspect of midwifery as miscarriages, stillbirths, maternal and neonatal deaths can occur. These experiences are incredibly difficult for the families involved, as well as for the midwives who, since ancient times, have traditionally worked with families from “womb to tomb” [2].

The attitudes toward death in midwifery are complex, shaped by personal beliefs, professional experiences and training, cultural backgrounds, and sociodemographic factors [3,4]. One prevailing attitude among midwives is a profound respect for the sanctity of life, which extends to a dignified acknowledgment of death [5]. Midwives often view death as an inevitable part of human life, understanding that their role encompasses providing comfort and support not only during birth but also during moments of loss [6]. This perspective fosters a sense of resilience and acceptance, enabling midwives to find their way through emotionally challenging situations with grace and empathy.

Emotional intelligence refers to the skillful ability of recognizing, understanding, and handling one’s own and others’ emotions effectively. Often, this involves considering one’s own reaction in the same situation and empathizing with others’ feelings [7]. Emotional intelligence plays a pivotal role in how midwives perceive and respond to death, as it equips them with the skills necessary to navigate the complexities of end-of-life care. Midwives with high emotional intelligence demonstrate heightened self-awareness, enabling them to recognize their emotional triggers and biases when confronted with death. This self-awareness fosters emotional resilience, allowing midwives to maintain composure and provide compassionate care in the face of adversity. Moreover, midwives with high emotional intelligence possess strong empathetic abilities, which facilitates them to connect deeply with grieving families and offer meaningful support tailored to their individual needs [8,9]. Considering that attitudes toward death and emotional intelligence are deeply intertwined in the practice of midwifery, where a compassionate and supportive care environment must be cultivated, undergraduate, postgraduate, and lifelong learning programs must foster open dialogues surrounding death, incorporate evidence-based training, and enhance emotional intelligence so that midwives can be empowered to provide end-of-life care with confidence and sensitivity [10,11,12,13]. Finally, providing opportunities for reflection and peer support may help midwives process their emotions, mitigate burnout, and uphold the highest standards of care for the families [14,15]. Midwifery care that encloses both the joys and sorrows that accompany the miracle of life ensures sustainability.

The relationship between personality traits and attitudes toward death includes associations among psychological, professional, and sociocultural dimensions [16,17]. Experiences around death can be emotionally taxing and challenge midwives’ professional resilience, and thus, understanding these correlations is of great importance for providing psychological support to midwives and ensuring their wellbeing, which in turn, affects the quality of care provided to women, neonates, and their families. Personality traits, as described by the Big Five personality traits model [18], include openness, conscientiousness, extraversion, agreeableness, and neuroticism. Each of these traits can profoundly impact how midwives perceive and cope with death. For example, midwives with high levels of openness may be more willing to explore and understand their feelings about death and seek out support or educational resources to cope with their experiences. They might be more adaptable to different coping strategies and open to discussing their experiences, which can aid in processing grief [19,20].

As for justice beliefs among midwives, these are deeply embedded in their practice. Midwives advocate for equitable access to care, aiming to reduce disparities in maternal and neonatal outcomes. They strive to ensure that all women, regardless of socioeconomic status, race, or location, have access to quality maternity care [21]. Additionally, central to midwifery care is the respect for women’s autonomy and the right to make informed choices about themselves, free from coercion [22], as well as the provision of culturally competent care. Midwives are trained to provide care that is sensitive to the cultural values and beliefs of the women they serve, promoting an inclusive approach to healthcare [23]. Furthermore, as part of their commitment to justice and equity, midwives often find themselves advocating for women’s rights within the healthcare system and society at large. This includes supporting reproductive rights, fighting against obstetric and interfamily violence, and challenging policies that restrict women’s choices and self-determination. Concisely, midwives are at the forefront of the healthcare system as active supporters of justice, equity, and human rights and must put a sustained effort into managing numerous challenges, such as legislative barriers, limited resources, and systemic biases within healthcare systems and cultures [24].

In the context of investigating the midwifery labor force in this study, it is appropriate to consider the framework of midwifery services in Greece. In Greece, midwifery services are primarily guided by the Ministry of Health, which establishes regulations, standards, and policies regarding midwifery practice to ensure the safety and quality of provided care. Midwives work in a variety of settings, including, for instance, hospitals, health centers, assisted reproduction units, family planning centers, and home birth settings, depending on the preferences and needs of the women they serve. Midwifery education in Greece consists of direct-entry programs, exclusively, and involves a comprehensive 4-year university program leading to a bachelor’s degree [25]. It typically follows European recommendations and directives; thus, the education and training of midwives in Greece are usually aligned with the standards set by the European Union (EU). Assistant nurse midwives undergo a two-year basic training program at Vocational Training Institutes. These programs are designed to provide students with the knowledge and skills necessary to assist midwives and other HPs in maternity care settings. Their role is ancillary, and they do not work independently. This specialty has existed in Greece since 2013, currently resulting in a limited presence of assistant nurse midwives in the workforce [26].

The conceptualization of this study was based on “The National Bereavement Care Pathway (NBCP) for Pregnancy and Baby Loss” launched in 2017 in England. As part of the NBCP, HPs provide bereavement care to those who have experienced miscarriage, termination of pregnancy due to fetal abnormalities, stillbirth, neonatal death, and sudden unexpected infant death. This initiative seeks to improve the quality of and reduce inequities in bereavement care provided by HPs [27,28]. In reviewing the existing literature, we found several studies that examined HPs’ attitudes toward death [3,29,30,31,32,33,34,35,36]. However, to the best of our knowledge, only one Greek study [37] has included midwives, among other HPs, working in Neonatal Intensive Care Units. Therefore, it is observed that Greek midwives remain an underexplored territory. By focusing our study exclusively on Greek midwives, we aim to fill this gap and contribute new understanding to the field by investigating the relationship between attitudes toward death with psychological constructs such as emotional intelligence, personality, resilience, and justice beliefs among midwives in Greece. Our research seeks to shed light on the attitudes toward death of Greek midwives working in various settings and enhance our understanding of the factors influencing them. The findings likely to emerge from these associations can potentially be used to develop targeted interventions and support mechanisms to improve midwives’ well-being and professional resilience and the quality of end-of-life and bereavement care they provide to women, newborns, and families.

## 2. Materials and Methods

### 2.1. Study Design and Ethical Considerations

This is a descriptive cross-sectional study conducted from September 2020 to September 2022. Participants were recruited from (a) four public tertiary hospitals, (b) primary networks pertaining to two out of the seven Regional Health Authorities in Greece, and (c) via email, if they were independent HPs from the authors’ network. Ethics approval was granted by the Committee of each hospital and Regional Health authority as follows:

- “ATTIKON” General University Hospital (ΙRB: A2/11/08-09-2020);
- “ALEXANDRA” General Hospital (IRB: 37/11/22-10-2020);
- General and Maternity Hospital “HELENA VENIZELOU” (IRB: 15056/07-07-2020);
- General Hospital of Nikaia—Piraeus “ST. PANTELEIMON” (IRB: 15/21/02-09-2020);
- 1st Regional Health Authority (IRB: 49856/11-11-2020);
- 2nd Regional Health Authority (IRB: Χ1936/22-06-2020).

### 2.2. Sample and Setting

Eligible participants were only midwives who worked in the aforementioned public hospitals and regional health authorities or as independent professionals in the prefecture of Attica, the capital of Greece. According to the inclusion criteria, the midwives should (a) be able to read and write in the Greek language in order to complete the questionnaires of the study and (b) have acquired a 3.5- or 4-year bachelor’s degree (technological educational institute or university). Assistant nurse midwives (graduated from a 2-year program) were excluded.

Out of a total of 500 midwives invited to participate in the study, 435 consented, resulting in a response rate of 87%. However, the final analysis was restricted to 348 participants who completed all five research instruments and were included in the analysis. The exclusion of 87 midwives was attributed to either incomplete questionnaire submissions or non-electronic submission, which was deemed insufficient for inclusion in the study. This sample size aligns with the established “10% rule”, with reference to the 3504 registered active members of the Athens Midwives Association at the beginning of the study in 2020. The recruitment process involved random selection from the Athens Midwives Association register. Athens, as the capital of Greece, hosts approximately 60% of the country’s midwives. The remaining 40% of midwives are registered in six other Midwifery Associations in different prefectures of Greece.

### 2.3. Data Collection

This study was based on the principles of quantitative methodology, with questionnaires as research instruments. The participants, after being informed about the research via a form, consented to their participation, which was completely based on the principles of the General Data Protection Regulation (GDPR).

Participants had the opportunity to fill in the questionnaires on paper and also online, when COVID-19 restrictions did not allow for distribution in person. Online data collection was facilitated through a secure platform (Microsoft forms) sanctioned by the university’s ethics committee. This platform employs several features and techniques to help ensure the validity of responses, including authentication, response limits, and data quality checks.

A rough estimate suggests that it took approximately 40 to 75 min for participants to complete all five research instruments, although the time it takes to complete the questionnaires can vary based on individual factors, such as reading speed, reading comprehension, and decision-making processes. Participants were requested to return the completed questionnaires within one week.

### 2.4. Research Instruments

In this study, midwives’ attitudes toward death were assessed using the Death Attitude Profile—Revised (DAP-R) [38]. It is a self-report questionnaire consisting of a total of 32 questions, using a 7-point Likert-type scale ranging from 1 (strongly disagree) to 7 (strongly agree). Five different subscales comprise this questionnaire. Specifically, fear of death, escape acceptance, approach acceptance, death avoidance, and neutral acceptance are analyzed [39]. The subscale “fear of death” (7 items) measures negative thoughts and feelings related to death. The subscale “death avoidance” (5 items) assesses the effort to avoid thinking about death. The “neutral acceptance” subscale (5 items) measures acceptance of death as a natural aspect of life, neither feared nor welcomed [29]. The “approach acceptance” subscale (10 items) assesses the view of death as an entrance to a better afterlife. The “escape acceptance” subscale (5 items) reflects the extent to which death is seen as a relief from suffering. The DAP-R has been translated into the Greek language and validated in the Greek population with acceptable reliability [40]. For each dimension of the DAP-R scale, the mean score can be calculated by dividing the total scale score by the number of items in each scale.

The Connor–Davidson Resilience Scale (CD-RISC) is a 25-question self-report instrument. Mental resilience was measured in the present study using this scale. Each question receives values from 0 to 4. The total score of the instrument captures the overall level of mental resilience. Resilience can be seen as a measure of the ability to cope with stress and, therefore, could be an important treatment target in stress reactions, anxiety, and depression. Treatment can modify a person’s resilience, with higher scores on the scale representing higher levels of overall improvement [41].

Participants’ emotional intelligence was measured using the Trait Emotional Intelligence Questionnaire—Short Form (TEIQue-SF) [42,43]. This particular questionnaire consists of 30 questions with values ranging from 1 to 7, where high values reflect high emotional intelligence. Although the TEIQue-SF is constructed to measure the overall emotional intelligence trait, according to Petridis (2009), four subscales can be derived, namely, emotionality, self-control, sociability, and well-being [42]. 

The Eysenck Personality Questionnaire (EPQ) was used to assess the personality traits of the study population [44], as was adapted by Demetriou for the Greek population [45]. The subscales measured by the EPQ are extraversion, neuroticism, and psychoticism, with the addition of the lie subscale. The instrument contains 84 close-ended questions (yes/no). The extraversion subscale represents sociability and impulsivity; hence, individuals in this dimension were identified as those who are energetic and enjoy social interactions as opposed to solitude. The neuroticism subscale denotes emotional instability and reactivity, with individuals scoring high on this subscale tending to be overly emotional, experiencing feelings of guilt, shyness, anxiety, and depression and generally having low self-esteem. The psychoticism subscale reveals personality traits such as being insensitive, distant, and irrational and lacking empathy for others [44,46]. Finally, the lie subscale was designed to assess dissimulation [46]. 

The perception of justice in the world was measured in this study using the Belief in a Just World scale (BJW). The BJW scale consists of 16 questions that assess the extent to which individuals believe that the world is fair and that people receive what they deserve. It is divided into two subscales, one concerning belief in a just world for oneself and the other about belief in a just world for others. Each subscale is further divided into beliefs in procedural and distributive justice [47].

In the present study, all included research instruments—DAP-R, CD-RISC, TEIQue-SF, EPQ, and BJW—had acceptable reliability coefficients (Cronbach’s alpha) above 0.70, indicating acceptable internal consistency for each scale [48]. The majority of previous Greek studies have demonstrated acceptable reliability coefficients for each instrument used. Malliarou et al. (2011) reported Cronbach’s alpha coefficients ranging from 0.65 to 0.86 for each subscale of the DAP-R (death avoidance = 0.85, neutral acceptance = 0.65, approach acceptance = 0.82, fear of death = 0.71, escape acceptance = 0.86) [40]. Tsigkaropoulou et al. (2018) found strong internal consistency for the CD-RISC, with a Cronbach’s alpha of 0.925 [49]. Stamatopoulou et al. (2016) reported high internal consistency for the TEIQue-SF total score, with a Cronbach’s alpha of 0.89 [43]. The study by Morfaki (2021) demonstrated Cronbach’s alpha coefficients ranging from 0.51 to 0.81 for each subscale of the EPQ (extraversion = 0.7153, neuroticism = 0.8129, psychoticism = 0.5114, lie scale = 0.6611) [46]. The reported reliability indices for BJW for Self and BJW for Others were 0.74 and 0.59, respectively, as demonstrated in the study by Tatsi and Panagiotopoulou (2021) [50].

### 2.5. Statistical Analysis

Quantitative variables were expressed as mean (±standard deviation) and median (interquartile range), while categorical variables were expressed as absolute and relative frequencies. The one sample Kolmogorov–Smirnov test was used to estimate the normality of the distribution of the quantitative parameters. The Spearman correlation coefficient (ρ) was used to investigate the correlation between two continuous variables. To identify factors independently associated with participants’ scores in DAP-R subscales, multiple linear regression was performed using a stepwise method (*p* for entry 0.05, *p* for removal 0.10). From the results of the linear regression analyses, adjusted regression coefficients (β) with standard errors (SE) and standardized coefficients (beta) were calculated. Multiple linear regression was conducted after all DAP-R subscales were logarithmically transformed, as the assumption of normal distribution was not satisfied. Internal consistency reliability was determined by calculating the Cronbach’s alpha coefficient. Scales with reliability equal to or greater than 0.70 were considered acceptable. All reported *p* values are two-tailed. Statistical significance was set at *p* < 0.05, and analysis was conducted using SPSS statistical software (version 26.0).

## 3. Results

The study included data from 348 midwives. The majority of the participants were female (92.8%), between 41–50 years of age (33.9%), married (68.4%), and had acquired a Master’s of Science (MSc) degree (49.1%). Additionally, 71.6% of the participants had children and, more specifically, 46.8% had two children. The mean time of total professional experience was 17.1 years (SD = 9.5 years), and the mean time of experience in the present department was 9.9 years (SD = 7.8 years). Participants’ demographic and socioeconomic characteristics are presented extensively in Table 1.

Τhe descriptive statistics of all the studied scales, as well as their internal consistency coefficients, Cronbach’s alpha, are presented in Table 2. The mean score was 3.5 (SD = 1.4) in the death avoidance subscale, 2.9 (SD = 0.8) in the neutral acceptance subscale, and 3.9 (SD = 1.2) in the approach acceptance subscale. In addition, the mean score was 3.3 (SD = 1.2) in the fear of death subscale and 4.6 (SD = 1.0) in the escape acceptance subscale. All DAP-R subscales had acceptable reliability coefficients, i.e., above 0.70, indicating acceptable internal consistency of the scale.

Greater emotional intelligence was significantly associated with higher scores in the escape acceptance subscale (Table 3). Furthermore, higher scores in the neuroticism subscale were significantly associated with higher scores in the death avoidance, approach acceptance, fear of death, and escape acceptance subscales. Higher scores in distributive justice beliefs for self and other subscales were significantly associated with lower scores on the subscale of death avoidance and approach acceptance.

After conducting multiple regression analysis, it was found that no factor was significantly associated with the neutral acceptance subscale. However, neuroticism was significantly associated with the remaining subscales of the DAP-R (Table 4). Finally, the subscale of distributive justice beliefs for self was significantly associated with the subscales of death avoidance and approach acceptance.

## 4. Discussion

Τhe present study investigated, for the first time, the relationship between attitudes toward death and emotional intelligence, personality, resilience, and justice beliefs among certified midwives. By including five different research instruments—DAP-R, CD-RISC, TEIQue-SF, EPQ, and BJW—we feel strongly that knowledge around this topic has been broadened, especially for the Greek midwifery labor force. 

In the present study, midwives reported lower levels of neutral acceptance (mean ± SD: 2.9 ± 0.8) compared to approach acceptance (3.9 ± 1.2) and escape acceptance (4.6 ± 1.0) on the DAP-R subscales. Lower scores in the neutral acceptance subscale suggests that midwives find it challenging to face death as a natural part of life. Additionally, higher scores in the approach and escape acceptance subscales means that midwives tend to perceive death as an entrance to a better afterlife and more as a relief from pain, respectively. These findings are consistent with a recent study of Israeli hemato-oncologists [29], a study by Black (2007) of physicians’ involvement with geriatric patients [30], and a Greek study of HPs, including midwives, working in neonatal intensive care units [37]. However, contradictory findings are reported in a study by Malliarou et al. (2011) [31] including palliative care nurses and in a study by Bouri et al. (2017) [32] including HPs providing pediatric palliative care, in which neutral acceptance received higher scores than the aforementioned subscales. When individuals exhibit low levels of neutral acceptance, it may indicate several psychological or emotional states regarding death, such as denial, existential concerns, or personal experience with death [51,52]. Denial about the inevitability of death can lead to avoiding thoughts or discussions about death, and existential concerns may reflect deeper struggles, such as finding meaning in the finite life on earth or dealing with the existential reality of non-existence after death. Furthermore, individuals who have had traumatic experiences with death or loss, such as the loss of a beloved person, especially in difficult circumstances, may find it harder to adopt a neutral stance toward death [52,53,54]. 

The results of the present study showed that greater emotional intelligence was significantly associated with higher scores on the escape acceptance subscale of the DAP-R, indicating that midwives may have more effective coping mechanisms for dealing with the concept of death and dying. This could lead to a more accepting attitude toward death, as death is perceived as an escape of suffering. Additionally, midwives with high emotional intelligence may have a greater capacity for empathy, allowing them to understand and resonate with the suffering of themselves or others [9,55]. Previous evidence from nursing students suggests that as students advance in their studies, they become less fearful of death [9], which is a finding that is important in terms of the design of academic programs. Moreover, Dooley et al. (2018) [56] explored final year undergraduate nursing and midwifery students’ perceptions of emotional intelligence, and although it was recognized that emotional intelligence was an important concept, the application of it to their curriculum was poorly defined. The participants acknowledged the emotional aspects of learning both in the classroom and in clinical education but lacked the skills and/or support to link coping strategies to emotional intelligence. Finally, in an attempt to create emotional control in a clinical environment, a number of students suppressed or denied emotions or distanced themselves from tasks and policies.

As for the correlation analysis between the DAP-R and the EPQ-R scales, we found that higher scores in the neuroticism subscale were significantly associated with higher scores in the death avoidance, fear of death, approach acceptance, and escape acceptance subscales. Midwives with high levels of neuroticism, characterized by emotional instability and reactivity, may experience stronger emotional reactions to death and may be more prone to feelings of guilt, shyness, anxiety, and depression in response to traumatic events [57]. In addition, they may have a harder time handling stress in acute situations [58]. Their coping mechanisms might include avoidance or excessive rumination in dealing with death-related situations, which could interfere with their professional functioning and personal well-being, impacting their ability to effectively provide care and support to patients and manage their own emotional health [37]. This correlation highlights the importance of understanding how personality traits, such as neuroticism, can influence the attitudes, emotions, and coping strategies of HPs. Addressing such factors can be crucial for supporting the well-being and resilience of midwives and improving the quality of midwifery care. 

In addition, regarding the correlation analysis of the DAP-R with the BJW scale, the results of the study revealed that higher scores in the distributive justice beliefs for self and others subscales were significantly associated with lower scores on the subscale of death avoidance and approach acceptance. Distributive justice beliefs refer to beliefs about fair outcomes, which could either concern oneself and be related to better life satisfaction and well-being or others and be associated with harsh social attitudes, including negative opinions of immigrants, people with illnesses, and the socioeconomically deprived [47]. Midwives who perceive the world as fair and believe that people generally receive what they deserve may be less likely to avoid thinking about death or view it as an entrance to a better afterlife. These results provide insights into how perceptions of fairness and justice in the world may interact with attitudes toward death. Previous evidence reports that the occurrence of traumatic events, such as the loss of a loved person, may reduce the individual’s belief in the world as just [58]. On the other hand, a recent study that included nursing students concluded that as students’ beliefs in a just world increased, they developed positive attitudes toward death [59]. Understanding the interplay between perceptions of fairness and attitudes toward death can help inform strategies for supporting midwives and other HPs in dealing with end-of-life care and related emotional challenges. This underscores the importance of addressing broader belief systems and worldviews in interventions aimed at enhancing coping mechanisms and emotional resilience in healthcare settings. Finally, the CD-RISC was not significantly associated with the DAP-R subscales.

While the presented findings are novel and provide valuable insights into midwives’ attitudes toward death, it is essential to recognize the inherent limitation associated with the reliance on self-report measures. Data obtained through self-report may not fully capture the subtle aspects of attitudes toward death, which individuals may not consciously acknowledge or may deny or actively avoid. Human psychology is intricate, and individuals may have subconscious or implicit responses to certain topics, such as death, that might not be fully revealed through self-reporting alone. The potential limitations of self-report measures underscore the need for a multifaceted approach in future research endeavors. Incorporating complementary methodologies, such as observational studies or qualitative interviews, could offer a more comprehensive understanding of the complex interplay of emotions and perceptions related to death among midwives. Another limitation of the study arises from the time required to complete the five research instruments, which could have potentially caused survey fatigue. This may have contributed to the situation in which 87 of the originally recruited midwives returned incomplete questionnaires or failed to submit them electronically. Moreover, the fact that the sample was derived from only one large city highlights the need for careful consideration when generalizing the results. Finally, the study did not collect any data regarding type of religion or religious beliefs [33]. Future studies may benefit from a holistic research design that combines quantitative self-report measures with qualitative insights or other methods that can capture implicit or subconscious aspects of attitudes toward this sensitive topic.

Furthermore, the integration of education on attitudes toward death into university curricula for HPs as a means of improving communication between HPs and patients and promoting HPs’ awareness of the potential impact of their own attitudes toward death on the well-being and perceived meaning of terminally ill patients and their families is of utmost importance [31,32]. The study by Schmit et al. (2016) [60] illustrates the benefits of education on end-of-life communication skills among physicians during residency and fellowship. Authors should discuss the results and how they can be interpreted from the perspective of previous studies and of the working hypotheses. The findings and their implications should be discussed in the broadest context possible. Future research directions may also be highlighted.

## 5. Conclusions

This study uncovered several key insights into the attitudes of Greek midwives toward death. Firstly, it found that many midwives struggle with accepting death as an inherent aspect of life, often viewing it as an avenue to escape suffering rather than a natural occurrence. Secondly, the study revealed that midwives generally exhibit heightened levels of neuroticism, suggesting they are more prone to emotional reactivity and tend to shy away from situations involving death. Lastly, our findings suggested that midwives who believe in a fair world, whether for themselves or others, were less inclined to avoid contemplating death or perceived it as a positive transition. Understanding these different attitudes and associated factors is important for addressing the emotional and psychological needs of midwives in the context of their provided care, particularly within the Greek midwifery labor force. These findings highlight the nuanced perspectives within the healthcare community. As we delve deeper into the complexities of end-of-life and bereavement care, understanding these diverse attitudes is crucial for providing comprehensive and empathetic support to both patients and healthcare providers.

## Figures and Tables

**Table 1:** Participants’ demographic and socioeconomic characteristics (N= 358).

Variables		N (%)
Gender	Male	25 (7.2)
	Female	323 (92.8)
Age (years)	20–30	48 (13.8)
	31–40	96 (27.6)
	41–50	118 (33.9)
	≥51	86 (24.7)
Educational level	Tertiary education	162 (46.6)
	Master’s degree	171 (49.1)
	Doctoral degree	15 (4.3)
Family status	Unmarried	78 (22.4)
	Married	238 (68.4)
	Divorced	25 (7.2)
	Widower	7 (2.0)
Number of children	0	99 (28.4)
	1	48 (13.8)
	2	163 (46.8)
	3	37 (10.6)
	4	1 (0.3)
Total professional experience (years), Mean (SD)		17.1 (9.5)
Professional experience in the present department (years), Mean (SD)		9.9 (7.8)

**Table 2:** Descriptive statistics for Death Attitude Profile—Revised (DAP-R) scale, Trait Emotional Intelligence Questionnaire-Short Form (TEIQue-SF), Eysenck Personality Questionnaire, Connor–Davidson Resilience Scale (CD-RISC), and Belief in a Just World scale (BJW).

Research Instruments	Measurements				
	Minimum	Maximum	Mean (SD)	Median (IQR)	Cronbach’s Alpha
Death Attitude Profile—Revised (DAP-R)					
Death avoidance	1.00	7.00	3.5 (1.4)	3.4 (2.2–4.6)	0.83
Neutral acceptance	1.00	6.80	2.9 (0.8)	2.8 (2.4–3.4)	0.71
Approach Acceptance	1.00	7.00	3.9 (1.2)	3.8 (3.2–4.6)	0.76
Fear of death	1.00	6.57	3.3 (1.2)	3.1 (2.6–4.0)	0.80
Escape Acceptance	1.40	6.70	4.6 (1.0)	4.7 (3.9–5.3)	0.77
TEIQue-SF					
	2.5	6.5	5 (0.6)	5.1 (4.6–5.4)	0.82
Eysenck Personality Questionnaire (EPQ)					
Psychoticism	0.0	13.0	4 (2.3)	4 (2–5)	0.71
Extraversion	4.0	19.0	14 (3.6)	14.5 (12–17)	0.77
Neuroticism	1.0	21.0	10.8 (4.4)	11 (7–14)	0.80
Lie	1.0	16.0	7.6 (3.2)	8 (5–10)	0.72
Connor–Davidson Resilience Scale (CD-RISC)					
	25.0	98.0	68.6 (12.5)	29 (27–31)	0.91
Belief in a Just World (BJW)					
Distributive Justice Beliefs for Others	4.0	28.0	15 (5)	15 (11–18)	0.78
Procedural Justice Beliefs for Others	4.0	23.0	12.6 (4.3)	12 (9–15)	0.74
Distributive Justice Beliefs for Self	4.0	25.0	16.8 (4.6)	17 (14–20)	0.78
Procedural Justice Beliefs for Self	9.0	28.0	19.6 (3.9)	20 (17–23)	0.75

**Table 3:** Correlation analysis of Death Attitude Profile—Revised (DAP-R) scale with Trait Emotional Intelligence Questionnaire Short Form (TEIQue-SF), Eysenck Personality Questionnaire, Connor–Davidson Resilience Scale (CD-RISC), and Belief in a Just World (BJW) scales.

		D (DAP-R)				
		Death Avoidance	Neutral Acceptance	Approach Acceptance	Fear of Death	Escape Acceptance
TEIQue-SF	ρ ^1^	0.08	0.01	−0.01	0.08	0.19
	*p* ^2^	0.168	0.948	0.976	0.149	0.001
EPQ						
Psychoticism	ρ	−0.08	−0.05	−0.02	−0.05	−0.04
	*p*	0.157	0.397	0.766	0.367	0.509
Extraversion	ρ	−0.08	−0.03	0.00	0.00	0.11
	*p*	0.140	0.546	0.994	0.975	0.048
Neuroticism	ρ	−0.13	0.06	−0.20	−0.24	−0.31
	*p*	0.019	0.262	<0.001	<0.001	<0.001
Lie	ρ	0.06	0.01	0.04	−0.08	−0.06
	*p*	0.314	0.924	0.416	0.141	0.309
BJW						
Distributive Justice Beliefs for Others	ρ	−0.17	−0.06	−0.14	0.06	−0.05
	*p*	0.001	0.277	0.008	0.306	0.409
Procedural Justice Beliefs for Others	ρ	−0.05	−0.03	−0.03	0.10	0.00
	*p*	0.361	0.560	0.561	0.061	1.000
Distributive Justice Beliefs for Self	ρ	−0.19	−0.03	−0.17	−0.03	−0.06
	*p*	<0.001	0.620	0.001	0.615	0.239
Procedural Justice Beliefs for Self	ρ	−0.08	−0.09	−0.05	−0.02	−0.06
	*p*	0.147	0.101	0.363	0.712	0.276
CD-RISC	ρ	0.02	0.02	−0.08	0.06	0.05
	*p*	0.659	0.782	0.130	0.316	0.369

^1^ ρ: for Spearman correlation coefficient. ^2^ *p*: for *p*-value.

**Table 4:** Multiple linear regression analysis results with dependent variables the DAP-R subscales in a stepwise method.

Dependent Variables	Independent Variables	β +	SE ++	B ‡	*p*
Death avoidance	Distributive Justice Beliefs for Self	−0.007	0.002	−0.171	0.002
	Neuroticism	−0.006	0.002	−0.150	0.005
Neutral acceptance	-	-	-	-	-
Approach Acceptance	Neuroticism	−0.006	0.002	−0.182	0.001
	Distributive Justice Beliefs for Others	−0.004	0.002	−0.141	0.009
Fear of death	Neuroticism	−0.008	0.002	−0.232	<0.001
Escape Acceptance	Neuroticism	−0.007	0.001	−0.303	<0.001

Note. Log transformations were conducted for the dependent variables. + regression coefficient ++ Standard error ‡ standardized coefficient.

## Data Availability

The original contributions presented in the study are included in the article, further inquiries can be directed to the corresponding authors.
